# Comparison of Drug-Coated Balloons and Drug-Eluting Stents in Primary Percutaneous Coronary Interventions for ST-Segment Elevated Myocardial Infarction: A Systemic Review and Meta-Analysis

**DOI:** 10.31083/j.rcm2306203

**Published:** 2022-05-31

**Authors:** Hu Su, Menghuan Li, Lijun Hao, Hui Wang

**Affiliations:** ^1^Department of Cardiology, The First Affiliated Hospital of Nanjing Medical University, 210000 Nanjing, Jiangsu, China; ^2^Department of Cardiology, Sir Run Run Hospital, Nanjing Medical University, 210000 Nanjing, Jiangsu, China

**Keywords:** drug-eluting stents, balloon, angioplasty, myocardial infarction, meta-analysis

## Abstract

**Background:**

The most optimal strategy for ST-segment elevation myocardial infarction (STEMI) between drug-eluting stents (DES) and drug-coated balloons (DEB) is still unknown. This meta-analysis aims to compare the short-term outcomes of both methods in patients with STEMI.

**Methods:**

We searched PubMed, Web of Science, EMBASE, and the Cochrane Library Databases for eligible studies with publication data from 2015 to Jan 2022. Four trials with 360 patients were included. The study was conducted by following the guidelines of the Preferred Reporting Items for Systematic Reviews and Meta-Analyses statements.

**Results:**

There were no significant differences in major adverse cardiac events between DCB and DES during 6 to 12 months of follow-up (RR 1.38, 95% CI: 0.65 to 2.93; *p* = 0.41). Similar risks of myocardial infarction (RR 0.48, 95% CI: 0.11 to 2.11, *p* = 0.33), all causes of death (RR 1.55, 95% CI: 0.32 to 7.62, *p* = 0.59), and target lesion revascularization (RR 1.29, 95% CI: 0.55 to 3.04, *p* = 0.55) were observed. The pooled results indicated that DCB was comparable to DES in terms of late lumen loss with a mean difference (MD) of –0.06 mm with significant heterogeneity (95% CI: –0.25 to 0.13, *p* = 0.54, $I^{2}$ = 85%). Subsequent subgroup analysis based on the study design revealed that late lumen loss was significantly lower in the drug-coated balloon group in randomized controlled trials (MD –0.16, 95% CI: –0.26 to –0.05, *p* = 0.003).

**Conclusions:**

Drug-coated balloons were associated with similar risks of MACE compared with drug-eluting stents in the setting of STEMI. However, a larger randomized controlled trial is required to confirm these observations.

## 1. Introduction 

Primary Percutaneous Coronary Intervention (PCI) is a major reperfusion strategy in patients with ST-segment elevation myocardial infarction (STEMI), and results in better outcomes than intravenous thrombolytic therapy [1]. Drug-eluting stents (DES) have been shown to reduce revascularization in STEMI patients compared to bare-metal stents (BMS) [2]. Nevertheless, DES can result in in-stent thrombosis and recurrent myocardial infarctions [3, 4]. Additionally, the metal scaffolding of stents may impair the endothelial and vasomotor function of coronary arteries [5].

Drug-coated balloons (DCB) are semi-compliant balloons containing antiproliferative agents that are effectively released when they dilate and come into contact with the walls of blood vessels [6]. In recent years, DCB has emerged as a novel technique of revascularization and showed tremendous benefits in patients with in-stent restenosis and stenosis of small coronary vessels.

Several studies have shown DCB to be a safe and effective treatment for STEMI [7, 8, 9]. A small sample study demonstrated that the DCB strategy was similar to DES when it comes to fractional flow reserve in the setting of STEMI during 9 months of follow-up [7]. Additional studies also showed comparable outcomes between DCB and DES regarding major adverse cardiac events (MACE), target lesion revascularization (TLR), and late lumen loss (LLL) [7, 8, 10].

However, an observational study derived from the AMI-DEB (drug-eluting balloon in ST-segment elevation myocardial infarction) trial found that DES was superior to DCB for MACE and LLL [11]. The most optimal strategy for STEMI remains unknown. Therefore, the purpose of this study was to compare the short-term efficacy of these two approaches in STEMI patients by conducting a meta-analysis.

## 2. Materials and Methods

### 2.1 Methods

This study was based on the PRISMA (Preferred Reporting Items for Systematic Reviews and Meta-Analyses) recommendations.

### 2.2 Study Protocol 

This study enrolled randomized control trials (RCTs) and an observation study comparing outcomes with DCB versus DES in the setting of STEMI. The follow-up period was at least 6 months. There were no limits on the sample sizes of the included studies.

The exclusive criteria were as follows: (1) studies assessing DCB’s efficacy for the patients with non-STEMI (NSTEMI), (2) studies comparing the outcome with DCB + BMS versus DES, (3) studies including planned DCB + stenting, (4) insufficient outcome data.

Informed consent were obtained from all subjects involved in these studies. All studies were performed in accordance with the Declaration of Helsinki.

### 2.3 Search Strategy 

Since the first study comparing DEB and DES was conducted in 2015; we searched PubMed, Web of Science, EMBASE, and the Cochrane Library Databases for eligible studies with publication data from 2015 to Jan 2022. The following search terms were used separately and in combination: “Drug-coated balloon”, “DCB”, “Drug-eluting balloon”, “DEB”, “paclitaxel-coated balloon”, “Drug-eluting stents”, “DES”, “acute myocardial infarction”, and “STEMI”. Additional filters, such as the article type and English language, were also used.

### 2.4 Selection Process and Data Extraction 

Two authors (HS and MHL) screened relevant studies independently. Studies were excluded based on their titles and abstracts. We reviewed the full text of all potentially eligible studies. In order to reduce intra-observer discrepancies, a consensus was necessary from both screening authors. The selection process was in strict accordance with the inclusive and exclusive criteria.

Data were extracted independently by the same authors. Prespecified information was formulated to enable extraction from eligible studies.

### 2.5 Study Outcomes

The primary outcome of this study was the incidence of MACE, which was the composite outcome of cardiac death, myocardial infarction (MI), and target lesion revascularization (TLR). The secondary outcome was defined as the incidence of each component of the composite endpoints. The angiographic outcome was late lumen loss (LLL) defined as the difference in minimal lumen diameter (MLD) at the same segment between post-angiography and follow-up.

### 2.6 Assessment of Study Quality and Risk of Bias 

The Newcastle Ottawa Scale (NOS) was applied to evaluate the quality of the cohort study. This scale assessed the selection of cohorts, comparability, and outcomes. A trial’s quality was considered to be high when the score was 7 or more estimated with the NOS. In addition, the risk of bias in RCTs was evaluated by using the Cochrane Collaboration’s Risk of Bias tool.

### 2.7 Statistical Analysis 

Risk ratio (RR) and 95% confident interval (95% CI) were estimated for binary outcomes, such as MACE, myocardial infarction, cardiac death, and TLR. Weighted mean difference (MD) and 95% CI were calculated for the continuous outcome. LLL was reported as a median value with an interquartile range in Vos *et al*. [7], a specific function algorithm was performed to calculate the mean and standard deviation [12, 13, 14, 15]. Estimates and 95% CIs were graphically presented using Forest plots [16]. $I^{2}$ statistics were calculated to examine the heterogeneity between studies. The fix effects model was applied to pool the effect size if $I^{2}$ > 50%. Otherwise, the random-eff model was used. A trial sequence meta-analysis (TSA) was conducted to evaluate the false positive (type 1 errors) and false-negative errors (type II errors) based on the current cumulative sample size. Review Manager software version 5.3 (2014, The Nordic Cochrane Centre, The Cochrane Collaboration, Copenhagen, Denmark), and TSA was conducted using TSA software (version 0.9.5.10 Beta, Copenhagen Trial Unit, Rigshospitalet, Denmark) to perform all the statistical analyses. Data was considered statistically significant when the *p*-value was < 0.05.

## 3. Results 

**Fig. 1.**
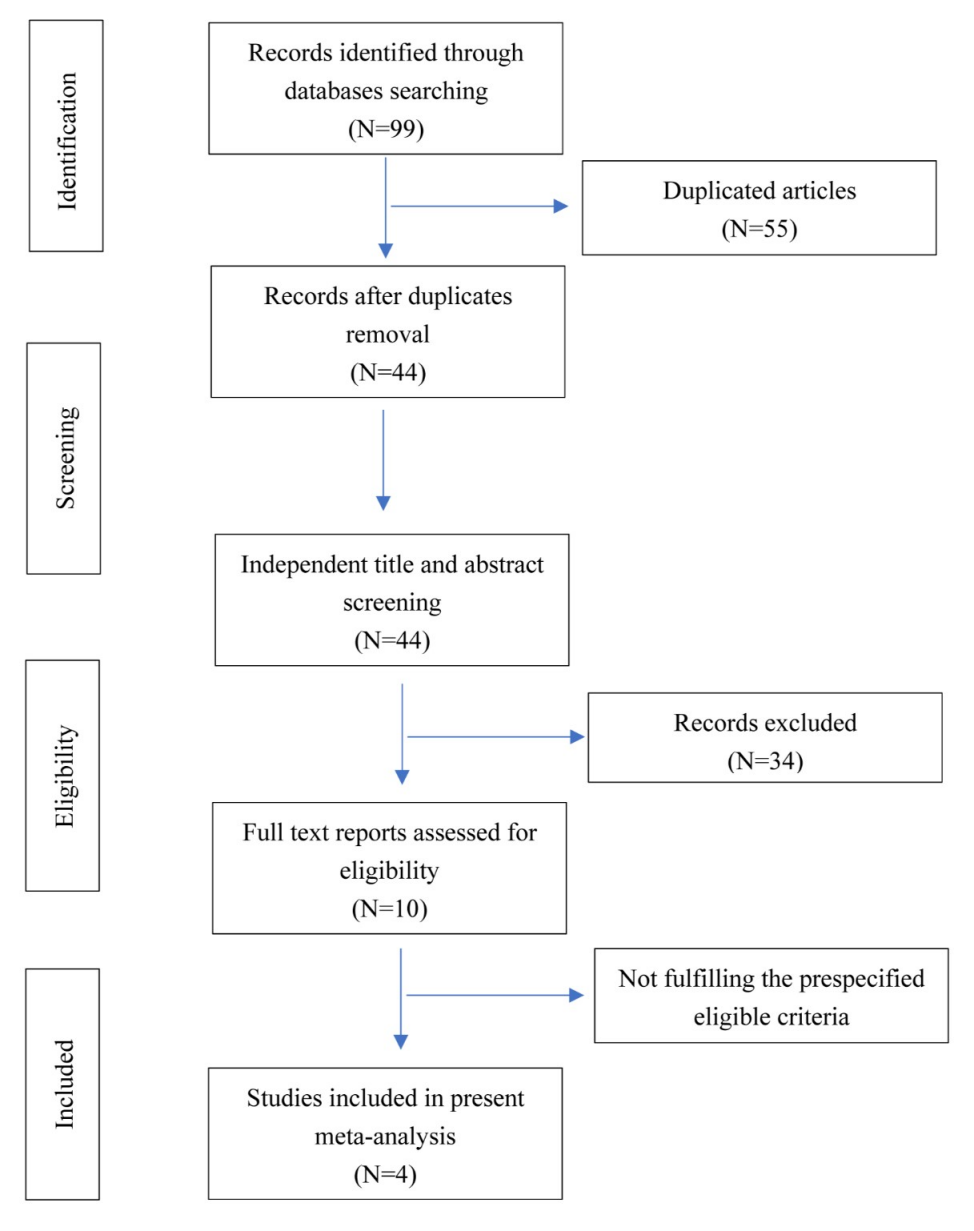
**Flow diagram**.

The study research and selection process are illustrated in Fig. 1.

A total of 99 studies were initially identified, among which, 44 articles were screened after removing duplicates. Finally, 4 studies were included in the present meta-analysis, including three RCTs and one observation cohort study. The general characteristics of the included trials are presented in Table 1 [7, 8, 10, 11].

**Table 1.** **General characteristics of included trials**.

Source	Country	Study type	Number of centers	Treatment										Follow-up period (months)	Bailout stenting (%)
				DCB					DES						
				Number of patients	DEB type	Age	HBP	DM (%)	Number of patients	Stent type	Age	HBP	DM		
Nijhoff 2015 [11]	Netherlands	non-RCT	2	40	Paclitaxel	57.9 ± 10.0	35%	12.50%	49	Paclitaxel	55.9 ± 9.7	30.60%	4.10%	12	10%
Gobić 2017 [10]	Croatia	RCT	not mentioned	37	Paclitaxel	57.2 ± 13.1	31.70%	4.90%	38	Sirolimus	54.3 ± 10.6	35.10%	10.80%	6	7.30%
Vos 2019 [7]	Netherlands	RCT	1	59	Paclitaxel	57.4 ± 9.2	30%	13%	61	Sirolimus/Everolimus	57.3 ± 8.3	32%	7%	9	18.30%
Good 2021 [8]	China	RCT	1	38	Paclitaxel	59 ± 11	22%	28%	42	not mentioned	56 ± 11	26%	35%	12	9.50%

RCT, randomized controlled trials; DEB, drug-eluting balloons; DCB, drug-coated balloons; HBP, high blood pressure; DES, drug-eluting stents; DM, diabetes mellitus.

**Fig. 2.**
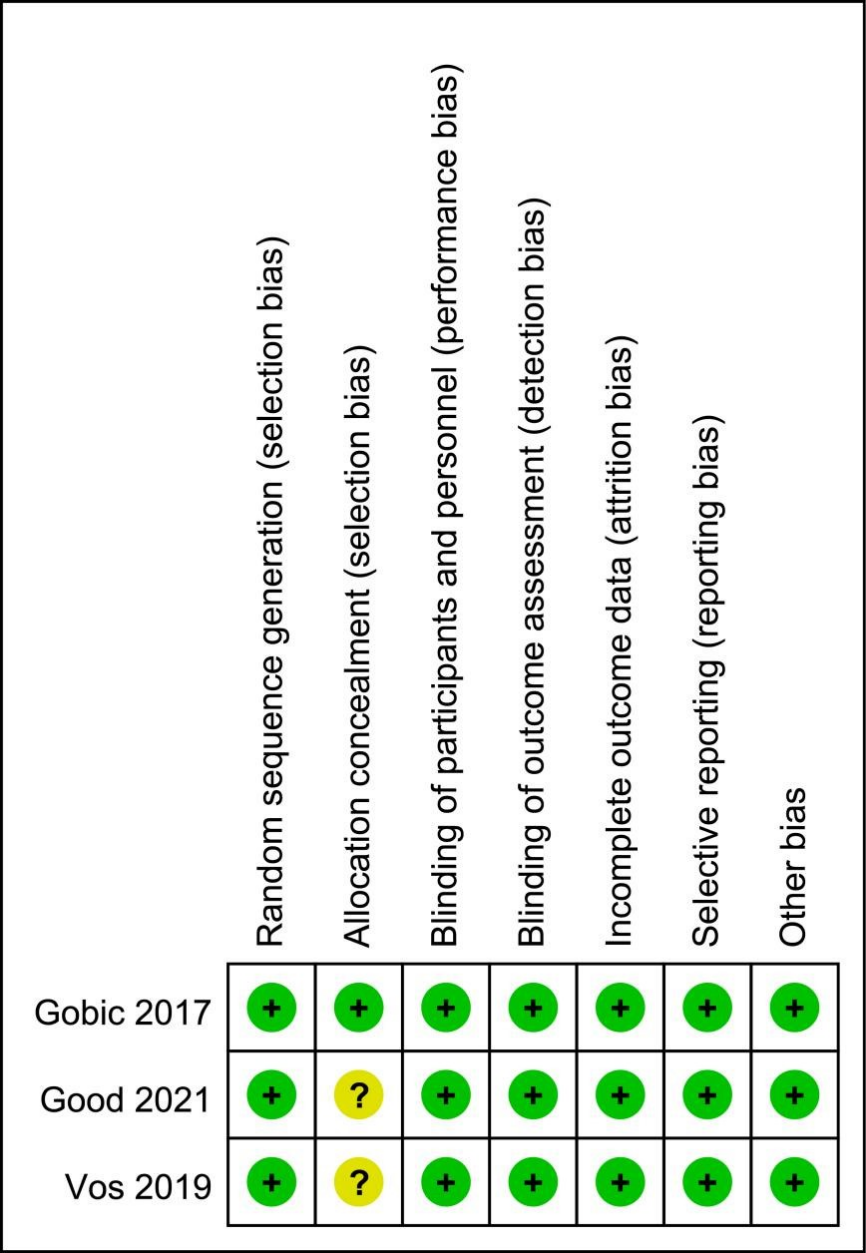
**Risk bias summary**.

**Fig. 3.**
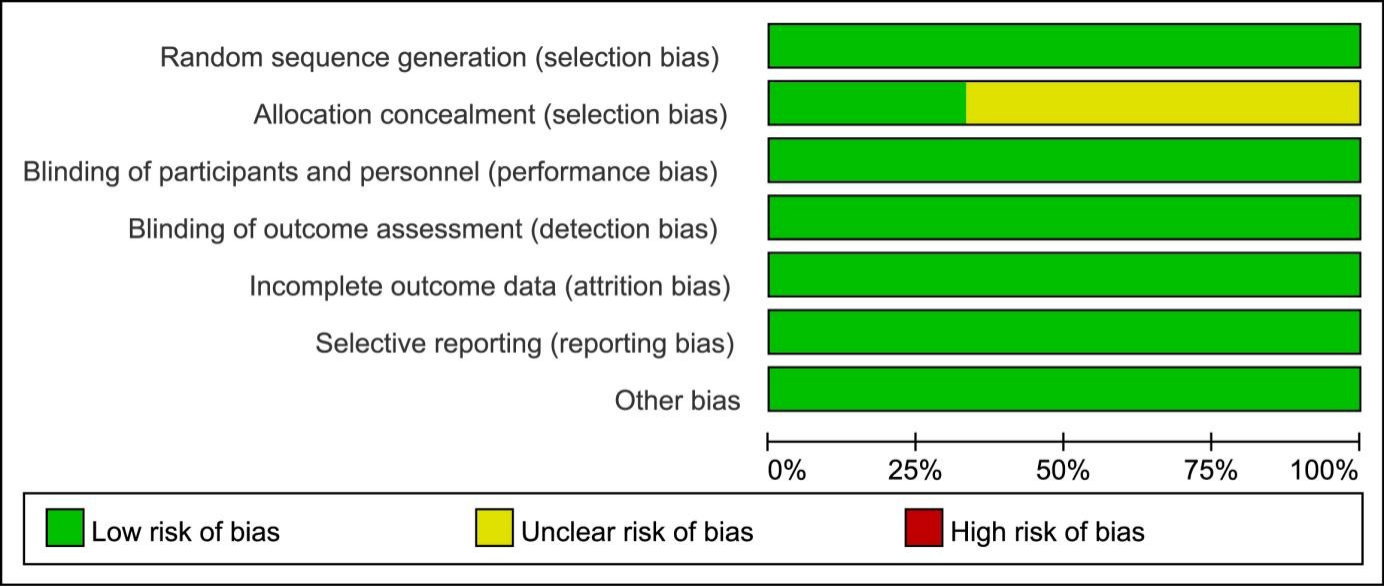
**Risk of bias graph**.

A total of 360 patients were analyzed. All the DCBs were coated with paclitaxel, and all trials had six to twelve months of follow-up. The observation trial in the present analysis was of high quality with eight scores evaluated by NOS (Table 2). The quality of all included RCTs were high based on strict research standards (Figs. 2,3).

**Table 2.** ** Quality assessment scale of Newcastle-Ottawa Scale (NOS) for non-randomized studies**.

Studies		Stars
Selection of cohort		★
	1. Representativeness of the exposed cohort.	★
	2. Selection of the non-exposed cohort.	★
	3. Ascertainment of exposure.	★
	4. Demonstration that outcome of interest was not present at the start of the study.	
Comparability		
	1. Comparability of cohorts on the basis of the design or analysis.	★ ★
Outcome		
	1. Assessment of outcome.	★
	2. Was follow-up long enough for outcomes to occur.	★
	3. Adequacy of follow-up of cohorts.	★
In total		8

### 3.1 Clinical Outcomes 

**Fig. 4.**
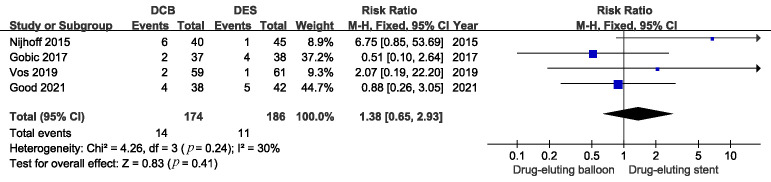
**Comparison of the risk of MACE between DCB and DEB**. MACE, major adverse cardiac events; DCB, drug-coated balloons; DES, drug-eluting stents.

The baseline characteristics, such as hypertension, diabetes mellitus, stroke, multi-vessels disease, and door to balloon time were similar between DCB and DES groups in all the included trials. A total of 360 patients were evaluated by MACE, 174 of whom were assigned to DCB treatment and the remainder were treated with DES. The results showed no significant difference in MACE incidence between DCB and DES during 6–12 months of follow-up. (Fig. 4. RR 1.38; 95% CI: 0.65 to 2.93; *p* = 0.41).

**Fig. 5.**
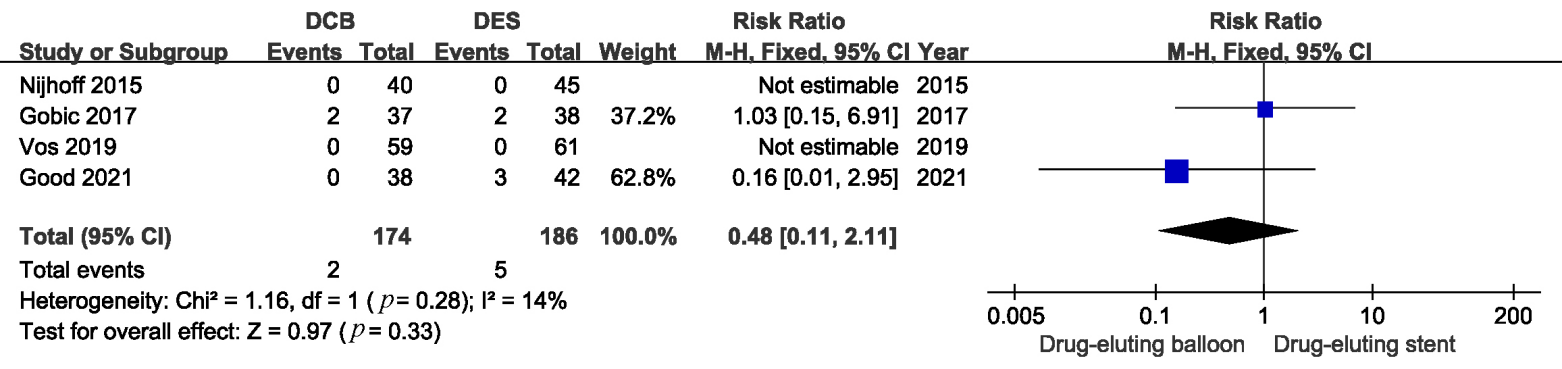
**Comparison of the risk of myocardial infarction between DCB and DES**. DCB, drug-coated balloons; DES, drug-eluting stents.

**Fig. 6.**
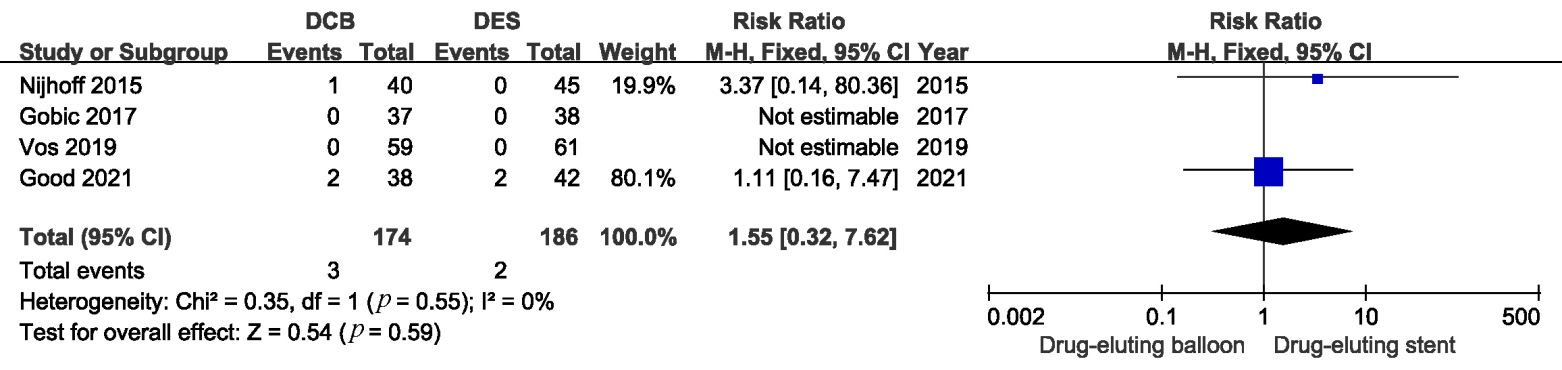
**Comparison of the risk of all causes of death between DCB and DES**. DCB, drug-coated balloons; DES, drug-eluting stents.

There were similar risks of myocardial infarction and all causes of death between the DCB and DES groups. (Figs. 5,6. MI: RR 0.48, 95% CI: 0.11 to 2.11, *p* = 0.33; Death: RR 1.55, 95% CI: 0.32 to 7.62, *p* = 0.59).

**Fig. 7.**
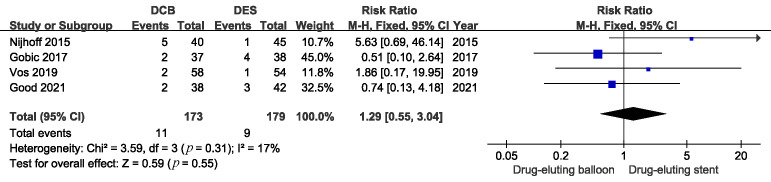
**Comparison of the risk of TLR between DCB and DES**. TLR, target lesion revascularization; DCB, drug-coated balloons; DES, drug-eluting stents.

In addition, the incidence of TLR was similar between the two groups (Fig. 7. Four trials with 372 patients, RR 1.29, 95% CI: 0.55 to 3.04, *p* = 0.55).

### 3.2 Angiographic Outcomes

**Fig. 8.**
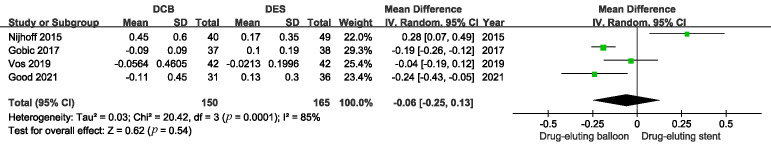
**Comparison of the risk of LLL between DCB and DES**. LLL, late lumen loss; DCB, drug-coated balloons; DES, drug-eluting stents.

Angiographical follow-ups were completed 6 to 12 months after the operation. LLL was reported in all the included studies involving 315 patients. The pooled results indicated that DCB was comparable to DES in terms of LLL with an MD of –0.06 mm with significant heterogeneity (Fig. 8. MD –0.06, 95% CI: –0.25 to 0.13, *p* = 0.54, $I^{2}$ = 85%).

**Fig. 9.**
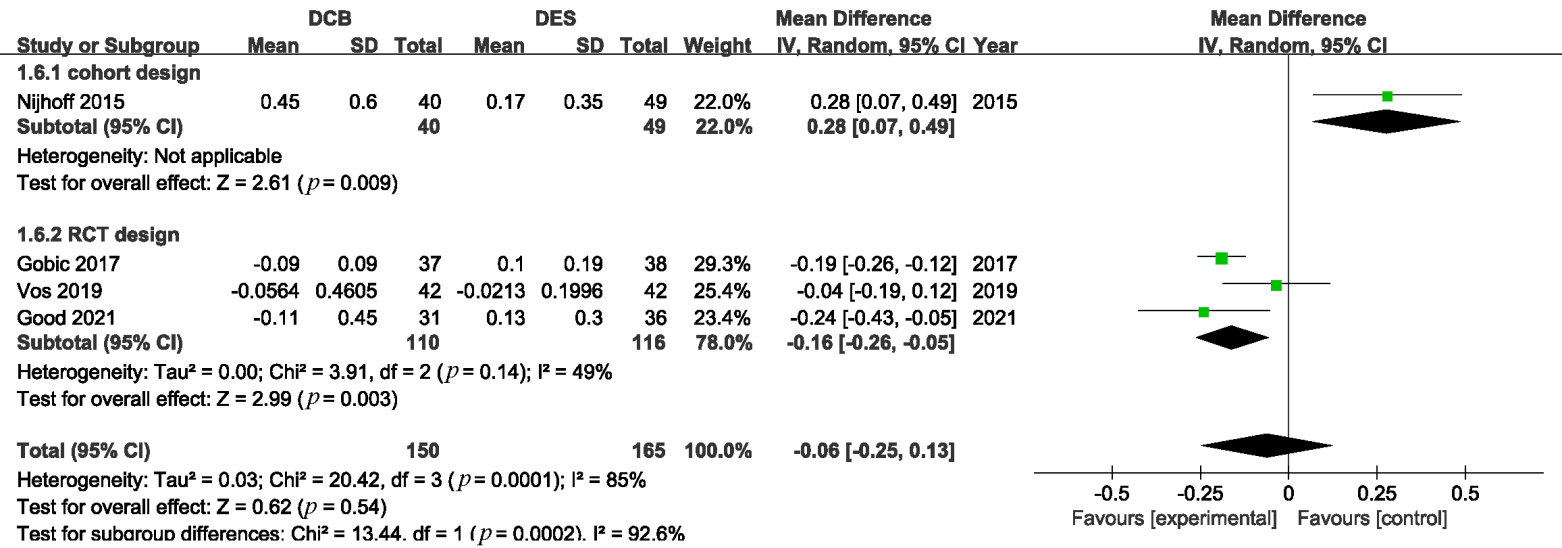
**Subgroup analysis according to the design of the trails**.

Subgroup analysis based on the study design revealed that LLL was significantly lower in the DCB group in RCTs (Fig. 9. MD –0.16, 95% CI: –0.26 to –0.05, *p *= 0.003).

### 3.3 TSA for TLR

**Fig. 10.**
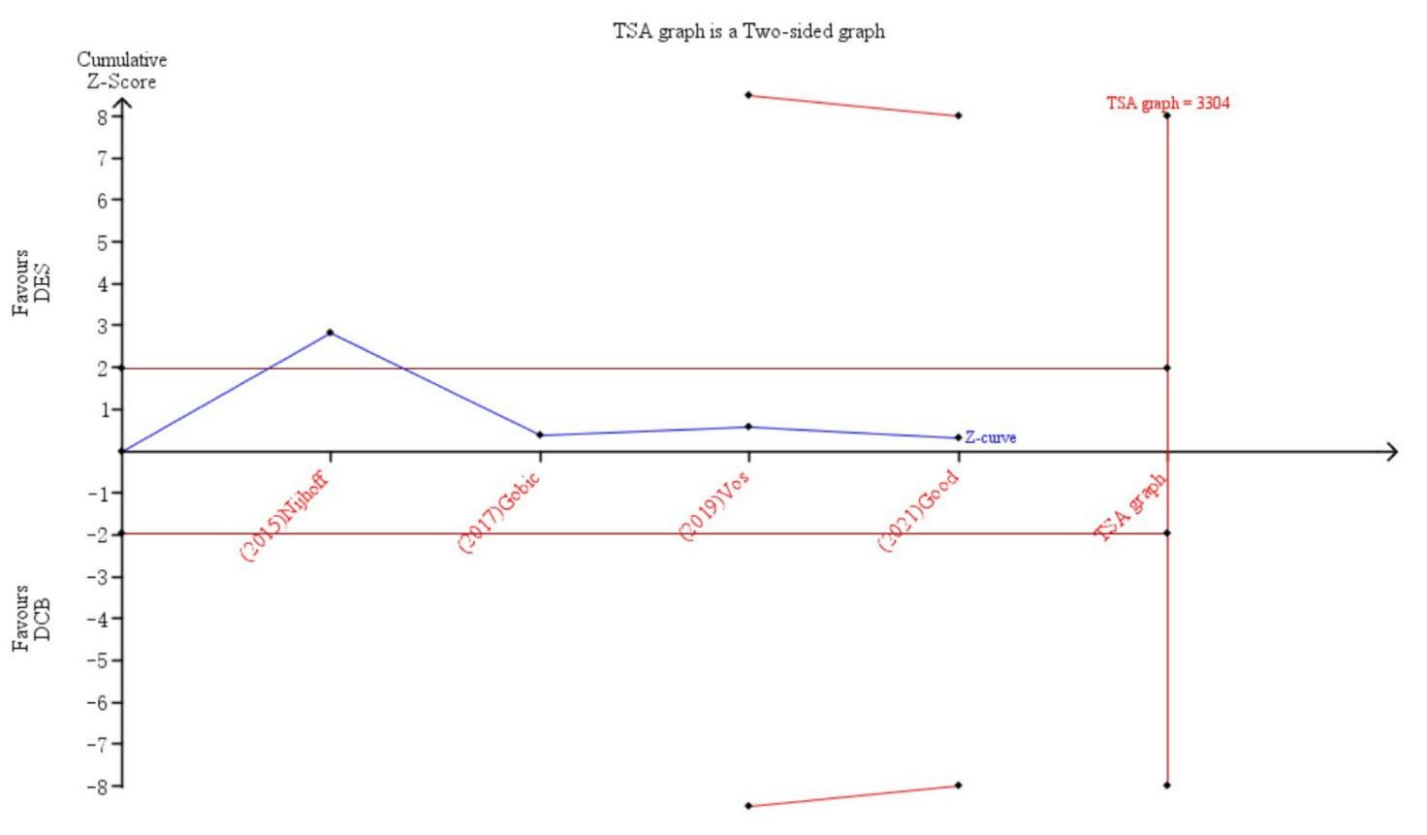
**TSA graph**.

TSA (Fig. 10) was performed to estimate the power of the conclusion derived from the four included trials. Predefining type 1 error as 5%, power as 80%, relative risk reduction as 30%, the required sample size was 3304 which meant that the current sample size was not large enough to fully confirm the conclusions. Although DCB was inferior to DES in terms of TLR based on these four trials, this result may represent a false negative conclusion.

## 4. Discussion

Our study, which includes three RCTs and one non-RCT, is the first meta-analysis to compare the short-term outcomes (clinical and angiographic outcomes) between DCB and DES in the setting of an STEMI, which differ widely from NSTEMIs in etiology, diagnosis, clinical manifestation, and therapeutic measures. Thus, choosing the most appropriate method of interventional therapy for STEMI is of great value. Statistical differences were not observed after careful analysis of clinical outcomes among enrolled patients, however, angiographic outcomes of lower LLL based on subgroup analysis may indicate the underlying advantages of DCB to maintain coronary vasomotor response and vessel shape. The current studies had a small sample size. Therefore, after assessing TSA for these trials, the conclusion that DCB was comparable to DES in STEMI may need to be confirmed in studies with a larger sample size.

Stent therapy replaces plain old balloon angioplasty (POBA) in STEMI because the latter is associated with recurrent ischemia, restenosis, re-occlusion of the target lesion, and a higher frequency of dissections [17]. Compared to uncoated stents, drug-eluting stents showed significant reductions in in-stent restenosis and in-stent late luminal loss. Several clinical trials have recently demonstrated that DES is superior to bare metal stents in STEMI patients because of improved long-term efficacy [18, 19, 20].

However, in the setting of STEMI, the significant risks of in-stent restenosis and late stent thrombosis associated with stent therapy also cannot be ignored [21]. Paclitaxel eluting stents tend to have a greater number of spatial distribution of uncovered and malapposed stent struts after implantation in patients with STEMI, as assessed by Optical Coherence Tomography (OCT) [22]. GaKu *et al*. [23] reported the prevalence of uncovered struts, fibrin deposition and inflammation were significantly higher in AMI patients compared with stable patients. In addition, vascular healing at implantation sites was significantly delayed when treated with DES as evidenced by an autopsy study [23].

Drug-coated balloons were introduced into clinical practice by combining drug coating technology with traditional balloon plasty. Antiproliferative drugs were delivered to local arterial tissue by a prolonged coated balloon angioplasty inflation, thus leaving no implanted material behind, thereby reducing late inflammation, allowing aggressive vascular remodeling, and shortening the duration of dual antiplatelet therapy [24]. DCB has been shown to be effective and safe in ISR treatment and is recommended in the latest ESC guidelines as a first-line treatment for ISR [25]. Studies show that more than 60% of ISR patients have an acute coronary syndrome, and about 10% of ISR patients may be at risk for sudden acute myocardial infarction [8, 26]. We believe that the treatment of ISR-induced MI will improve in the coming years with the increasing use of intravascular ultrasound imaging (IVUS).

We observed a significant degree of statistical heterogeneity for LLL. A DCB-AMI study that significantly affected the overall effect may be a reasonable for this observation. Considering the merits of non-RCT studies and the improvement of interventional techniques, a subgroup analysis was performed to observe the angiographic results in the RCTs group. Statistical significance between the two groups reveals the potential advantage of DCB. A lower LLL suggests a positive remodeling occurs in the vessel wall when more uniform antiproliferative drugs were delivered [26], which is a more precise and efficient method that decreases the release of the drug to the “blind zone”. The absence of metal bundles reduces the effect on the original anatomical structure of the blood vessels, as well as the coronary vasomotor response and vessel shape, and thus maintains normal blood flow to ensure enough oxygen supply to the myocardium [27, 28].

The incidence of MACE was 8% in the balloon group, 6% in the stent group, and the total number of MACE was 14 and 11 respectively during follow-up. We did not observe a statistically significant difference between them. Thus, we found that DCB treatment of STEMI is safe and effective, with good clinical outcomes during follow-up. Notably, the four studies we screened only used paclitaxel-coated balloons in the DCB group. Recent studies [29, 30] on different coating technologies and drug selection, involving zotarolimus, and everolimus DCB, have entered pre-clinical studies and demonstrated good safety and efficacy. In addition, the release of sirolimus nanoparticles in local coronary arteries in a pig model using a novel porous balloon release system achieved levels of long-term intraarterial drug therapy without significant systemic residual exposure [31].

Finally, the balloon has better maneuverability than the stent, it improves the immediate success rate of the operation, expands the diameter of the vessel after surgery, thus expanding its application. Dual antiplatelet therapy for 1 month after DCB is recommended based on current expert consensus [32]. Nevertheless, 6 months were needed after symptoms stabilize for patients with DES [33]. DCB therapy is, therefore, more appropriate for patients at a high risk for bleeding.

Interestingly, the current analysis showed that the risk of TLR in the DCB group was not significantly different compared with the DES group, but the risk was higher, with an RR of 1.29% (*p* = 0.540). We observed that more coronary artery dissection occurred in the DCB group after PCI, leading to more TLR. Previous studies have found that the presence of coronary dissection was predictive of subsequent ischemic events [34] which might explain why DCB was inferior to DES in terms of TLR based on these four trials. Additionally, different pharmacological mechanisms may be one of the reasons for the deviation of the TLR rate [35]. Compared to everolimus, paclitaxel seems to induce higher levels of acute inflammation and more chronic endometrial hyperplasia.

Similarly, a previous meta-analysis also showed there was no statistically significant difference in clinical and angiographic outcomes in AMI patients (including patients with NSTEMI) treated with DCB and DES [36]. Our study provides a more detailed analysis of STEMI lesions, which are mostly focal, soft, non-calcified occlusive lesions caused by the erosion or rupture of nonsignificant plaques in large vascular segments. On average, STEMI patients are younger than patients with stable coronary artery disease, where the lack of permanent implants can be a particular concern.

Overall, DEB is a sound treatment strategy for STEMI patients. Compared to DES, DCB is an acceptable alternative strategy with acceptable short-term effects and similar one-year clinical benefits. However, we should be cautious to interpret the results in consideration of the limitations of DCB technology. Secondary STEMI injury following plaque rupture is associated with varying levels of thrombotic burden [37]. The presence of thrombosis may interfere with the rapid and effective entry of antiproliferative agents into the coronary artery intima. Special attention should therefore be paid to adequate thrombus aspiration to avoid excessive interposed mural thrombus which reduces paclitaxel metastasis [38]. As with common BA, DCB is at risk for persistent residual stenosis, acute vasoconstriction, and detachment, which may require an emergency stent. Preclinical studies have shown significant differences in anti-restenosis efficacy between different DEBs due to differences in excipients and drug coating techniques, resulting in different paclitaxel release doses [6, 39, 40]. Therefore, we should be very cautious about extending our findings to the treatment of myocardial infarction. Subsequent larger randomized trials with appropriate clinical endpoints are needed to further elucidate the true benefits of DCB in coronary interventions.

## 5. Limitations

Several limitations of this study should be recognized. First, due to the small number of studies, patients, and events, our ability to detect differences in clinical outcomes is limited. Secondly, the presence of bailout stents prevents us from systematically assessing the impact of cross-treatment because most publications do not provide information on this intervention. Third, we did not evaluate important prognostic indicators such as restenosis, hemorrhage, and stent thrombosis because of the limited number of studies that included these events. Finally, pharmacokinetic differences between the DCB devices may cause an unpredictable influence when comparing DCB to DES. Meanwhile, newer DCB, such as sirolimus-coated balloons have shown favorable outcomes. Additional clinical trials are needed to confirm the advantages of DCB in patients with STEMI.

## 6. Conclusions

In this meta-analysis comprising 360 patients with STEMI, DCBs were associated with similar risks of myocardial infarction, all causes of death, and TLR compared with DES. While subgroup analyses indicated that DCB was superior to DES when it comes to LLL, TSA showed that the conclusion derived from the current trials may be incorrect. Therefore, a larger clinical trial is necessary to further confirm the role of DCB in patients with STEMI.

## Abbreviations

PCI, Primary percutaneous coronary; intervention; DES, drug-eluting stents; DCB, drug-coated balloon; STEMI; ST-segments elevation myocardial infarction; BMS, bare-metal stents; MACE, major adverse cardiac events; TLR, target lesion revascularization; LLL, late lumen loss; NSTEMI, non-STEMI; MI, myocardial infarction; RR, Risk ratio; CI, confident interval; MD, mean difference; TSA, Trial sequence meta-analysis; OCT, Optical Coherence Tomography intravascular; IVUS, ultrasound imaging.
